# Inflammatory Biomarkers as Mediators of the Effect Between High‐Dose Corticosteroid Therapy and Mortality in COVID‐19‐Related ARDS: A Causal Mediation Analysis

**DOI:** 10.1002/iid3.70427

**Published:** 2026-04-26

**Authors:** Katrijn Daenen, Anders Boyd, Virgil A. S. H. Dalm, Jilske A. Huijben, Sara C. M. Stoof, Lieuwe D. J. Bos, Diederik Gommers, Janke Schinkel, Eric C. M. van Gorp, Henrik Endeman

**Affiliations:** ^1^ Department of Intensive Care Erasmus University Medical Center Rotterdam the Netherlands; ^2^ Department of Viroscience Erasmus University Medical Center Rotterdam the Netherlands; ^3^ Stichting HIV Monitoring Amsterdam the Netherlands; ^4^ Infectious Diseases, Amsterdam University Medical Centers, Amsterdam Medical Center, University of Amsterdam Amsterdam the Netherlands; ^5^ Amsterdam Institute for Infection and Immunity Infectious Diseases Amsterdam the Netherlands; ^6^ Department of Immunology Erasmus University Medical Center Rotterdam Rotterdam the Netherlands; ^7^ Department of Internal Medicine, Division of Allergy & Clinical Immunology Erasmus University Medical Center Rotterdam Rotterdam the Netherlands; ^8^ Department of Intensive Care Amsterdam University Medical Centers, Academic Medical Centre, University of Amsterdam Amsterdam the Netherlands; ^9^ Laboratory of Experimental Intensive Care and Anesthesiology Amsterdam University Medical Centers, Academic Medical Centre, University of Amsterdam Amsterdam the Netherlands; ^10^ Department of Medical Microbiology & Infection Prevention Amsterdam University Medical Centers, University of Amsterdam Amsterdam the Netherlands; ^11^ Department of Internal Medicine Erasmus University Medical Center Rotterdam the Netherlands; ^12^ Department of Intensive Care OLVG Amsterdam the Netherlands

**Keywords:** biomarker, COVID‐19, inflammation, mediation, SARS‐CoV‐2

## Abstract

**Background:**

Treatment guidelines for acute respiratory distress syndrome (ARDS) recommend the use of high‐dose corticosteroids based on their anti‐inflammatory effects, yet the efficacy of this therapeutic strategy in patients with COVID‐19 related ARDS is inconclusive. To more thoroughly understand the mechanism of action for high‐dose corticosteroids in patients with COVID‐19‐related ARDS, we hypothesized that the reduction of inflammatory markers induced by high‐dose corticosteroids would reduce mortality (i.e., inflammation as a mediator).

**Design and Methods:**

In this retrospective cohort study, we included patients with COVID‐19‐related ARDS admitted to the intensive care unit (ICU) of an academic hospital in Rotterdam, the Netherlands between March 2020 and June 2022. High‐dose corticosteroids were defined as > 6 mg of dexamethasone/day or equivalent. Biomarkers included C‐reactive protein (CRP), d‐dimer, ferritin, leukocyte count, interleukin‐6 (IL‐6), lactate dehydrogenase, and neutrophil‐to‐lymphocyte ratio (NLR). Using a causal mediation framework, we estimated the average mediation effect (AME) and % mediation for each biomarker, allowing to quantify the degree to which continuous levels of inflammatory markers mediate the association between receiving high‐dose corticosteroids and mortality. The models used in this framework accounted for time‐varying treatment/mediation with inverse probability of treatment weights, determined from the covariates PaO_2_/FiO_2_ ratio, sex at birth, age, NLR, and ICU length of stay.

**Results:**

Of the 327 patients included, 122 (37.3%) received high‐dose corticosteroids. The risk of death was higher in patients who did vs. did not receive high‐dose corticosteroids [incidence rate = 0.54, 95% confidence interval (CI) = 0.42–0.71 and 0.21, 95% CI = 0.15–0.29/person‐month, respectively; *p* < 0.001]. This effect remained in most analyses accounting for time‐varying treatment/mediation. The association between high‐dose corticosteroids and mortality was reduced (i.e., mediated) with lower levels of CRP (AME = −0.006, 95% CI = −0.011, −0.002; %‐mediation = −82.0%), d‐dimer (AME = −0.002, 95% CI = −0.005, −0.001; %‐mediation = −33.1%), and IL‐6 (AME = −0.002, 95% CI = −0.004, −0.001; %‐mediation = −25.5%). There was no evidence of mediation for other biomarkers.

**Conclusion:**

CRP, d‐dimer, and IL‐6 mediated the association between high‐dose corticosteroids and mortality. When inflammation was reduced, the deleterious effect of high‐dose corticosteroids was eliminated.

**Abbreviations**
APACHE: acute physiology and chronic health evaluation
ARDS: acute respiratory distress syndrome
BMI: body mass index
CI: confidence interval
COVID‐19: coronavirus disease 2019
CRP: C‐reactive protein
FiO2: fraction of inspired oxygen
ICU: intensive care unit
IL‐6: interleukin‐6
IPTW: inverse probability of treatment weights
IQR: interquartile range
kPA: kilopascal
LDH: lactate dehydrogenase
NLR: neutrophil‐to‐lymphocyte ratio
OR: odds ratio
PaO2: partial pressure of oxygen in arterial blood
PCR: polymerase chain reaction
P/F ratio: PaO₂/FiO₂ ratio
SARS‐CoV‐2: severe acute respiratory syndrome coronavirus 2
SOFA: sequential organ failure assessment

## 1 Background

Acute Respiratory Distress Syndrome (ARDS) is a severe condition of the lungs that is associated with a high risk of mortality [1]. Infection with severe acute respiratory syndrome coronavirus 2 (SARS‐CoV‐2) can lead to a constellation of flu‐like symptoms, referred to as the coronavirus disease 2019 (COVID‐19), and in some cases, ARDS [2, 3, 4]. In fact, most patients with COVID‐19 who were admitted to the intensive care unit (ICU) had ARDS [5].

ARDS is characterized by acute hyper‐reactivity of the immune system with uncontrolled activation of immune cells and release of cytokines [6, 7]. In patients with COVID‐19‐related ARDS, several peripheral, immune‐inflammatory markers in the blood severely increase and include C‐reactive protein (CRP), leukocyte count, neutrophil‐to‐lymphocyte ratio (NLR), interleukin‐6 (IL‐6), d‐dimer, and lactate dehydrogenase (LDH) [8, 9]. The excessive release of these cytokines in patients with COVID‐19‐related ARDS is strongly associated with an increased risk of multiple organ failure and mortality [10]. Reducing such excessive inflammation would then seem necessary to decrease the risk of severe morbidity and mortality.

Corticosteroids are commonly used to treat patients with non‐COVID‐19‐related ARDS owing to their strong anti‐inflammatory effects [11]. In patients with COVID‐19 who receive oxygen therapy or invasive mechanical ventilation, their use as first‐line immunosuppressive therapy, administered as 6 mg of dexamethasone per day for 10 days or an equivalent corticosteroid, has clearly led to reduced mortality [12, 13]. However, in cases of further clinical deterioration, higher doses of corticosteroids are sometimes administered in an attempt to reduce the risk of death. Hypothetically, higher doses could reduce hyper‐inflammation, thereby improving patient outcomes.

The effects of high‐dose corticosteroid therapy on mortality could be mediated by specific immune‐inflammatory pathways. Hence, the overall aim of this study was to assess the mediating role of inflammatory biomarkers on mortality when high‐dose corticosteroid therapy is administered to patients admitted to the ICU with COVID‐19‐related ARDS. We hypothesized that high‐dose corticosteroids would reduce mortality in COVID‐19‐related ARDS patients through suppression of inflammatory biomarkers, with decreasing inflammation serving as a mediator between high‐dose treatment and improved survival. To this end, we conducted an analysis using a causal mediation framework in which the mediating effects of various inflammatory biomarkers on the association between high‐dose corticosteroid therapy and mortality were assessed.

## 2 Methods

### 2.1 Study Design and Population

We conducted a retrospective single‐center cohort study in the Netherlands. We analyzed data from patients with COVID‐19‐related ARDS who were admitted to the ICU of the Erasmus University Medical Center (Rotterdam, the Netherlands) between January 4, 2020, and December 31, 2022. In this study, patients were included if they were at least 18 years of age, had either a Polymerase Chain Reaction (PCR)‐confirmed SARS‐CoV‐2 infection or high clinical suspicion of COVID‐19, and had available data on inflammatory biomarkers. Patients who died or were discharged within 2 days after admission were excluded from the study.

The study was approved by the local Medical Ethics Review Committee under protocol number MEC‐2022‐0297 and conducted in accordance with the principles of the Declaration of Helsinki. Due to the urgency of conducting research in patients with COVID‐19, an exemption for consent was approved. An opt‐out informed consent procedure was used instead, and patients who objected to participation were excluded from the study (MEC‐2022‐0297).

### 2.2 Data Collection

Data were extracted from electronic health records. Patient data, including demographics, body mass index, vital signs, comorbidities, and laboratory tests, were collected from the day of ICU admission until hospital discharge or death. The APACHE‐IV predicted mortality score was extracted upon admission and the SOFA score was extracted daily. P/F ratios were calculated using the PaO_2_ and FiO_2_ measurements collected closest to 8 a.m. on the same day.

### 2.3 Study Variables in Mediation Analysis

#### 2.3.1 Study Exposure

High‐dose corticosteroid therapy was the exposure in analysis and was defined as treatment with > 6 mg of dexamethasone per day or equivalent as indicated during ARDS (Supporting Table 1). High‐dose corticosteroid therapy could be administered at any time from ICU admission until discharge from ICU and thus was considered as a time‐updated exposure.

Initially, the decision to commence high‐dose corticosteroid treatment was made by the attending physician at the ICU following multidisciplinary discussion. From April 7, 2021, this decision was based on protocols established at the Erasmus MC using information from general ARDS guidelines. The protocol recommended commencing high‐dose corticosteroid therapy (i.e., 1000 mg methylprednisolone during 1–3 days) for any patient classified as COVID‐19 stages IIb and III if the diagnosis of moderate/severe ARDS was < 14 days after ICU admission. COVID‐19 stages were derived from Siddiqi et al [14] and were based on the duration of illness, specific respiratory indicators (e.g., PaO_2_/FiO_2_ ratio), imaging findings, and the degree of systemic inflammation.

#### 2.3.2 Study Outcome

All‐cause mortality was the study outcome in this study. Patients were monitored from the moment of admission to the ICU until the time of hospital discharge, and thus all deaths reported were in‐hospital.

#### 2.3.3 Study Mediators

Levels of inflammatory biomarkers were the study mediators and were considered individually, and not collectively, in this study. We selected biomarkers of inflammation that are known to be correlated with disease severity and mortality in patients with COVID‐19, some of which are also involved in coagulation and endothelial injury [8, 9]. The biomarkers were as follows: CRP, d‐dimer, ferritin, leukocyte count, IL‐6, LDH, and NLR.

Biomarkers were measured using standard laboratory assays during the follow‐up period and were collected at the discretion of the ICU medical staff as part of routine care. Blood samples were obtained at the ICU and centrifuged (3000 N Relative centrifugal force) at room temperature for 5 min. The serum was analyzed on a routine analyzer (Roche Cobas 8000 system, Roche Diagnostics; Almere, the Netherlands). CRP and d‐dimer levels were measured using a turbidimetric method (C502 Cobas assay [15]) and ferritin and IL‐6 were measured using electro‐chemi luminescent immunoassays (E801 Cobas assay [16]). Leukocyte counts were determined using Sysmex automated cell counters, while May–Grünwald/Giemsa staining and microscopic examination were employed for visual confirmation. LDH levels were quantified through enzymatic analysis.

#### 2.3.4 Statistical Analysis

Baseline was defined as the date of ICU admission. Follow‐up began at baseline and continued until hospital discharge or death, whichever occurred first. We reconstructed the dataset such that baseline and follow‐up visits occurred in daily intervals. Time‐updated biological values were those collected closest to 8 a.m. of the calendar day. A patient was considered exposed to high‐dose corticosteroids the calendar day on which they commenced therapy. High‐dose corticosteroid exposure was considered present on every day after commencement until last follow‐up visit, as we assumed that any effect on mortality induced by high‐dose corticosteroid therapy would continue throughout follow‐up.

Baseline variables were summarized using medians and interquartile range (IQR) for continuous variables and counts and percentages for categorical variables. These variables were compared between patients who ever vs. who never received high‐dose corticosteroids using a Mann–Whitney *U* test for continuous variables and Pearson's *χ* ^2^ test for categorical variables.

To assess the determinants of commencing high‐dose corticosteroids, the probability of having high‐dose corticosteroids was modeled at each day using logistic regression with a random‐intercept to account for variation between patients at baseline. For this analysis, follow‐up was additionally censored after the first day on which high‐dose corticosteroids were given. Variables that were of clinical relevance or known predictors of both high‐dose corticosteroid treatment and mortality in critically ill COVID‐19 patients [10, 17, 18, 19, 20, 21, 22, 23, 24, 25, 26] were included individually in the model. We selected variables based on expert consensus from a panel of internists and intensivists who relied on both their clinical experience during the COVID‐19 pandemic and relevant literature. Univariable odds ratios (OR) comparing the odds of receiving high‐dose corticosteroids across levels of covariables and their 95% confidence intervals (CI) were calculated from this model. A multivariable model was constructed using a backward‐stepwise approach, whereby all variables with a *p* < 0.05 in univariable analysis were added to a full model and variables with a *p* > 0.10 were sequentially removed. The variables age and sex at birth were forced in the multivariable model.

To investigate the mediating effect of biomarkers on the association between high‐dose corticosteroid therapy and all‐cause mortality, we performed an analysis using a causal mediation framework based on the method from Imai, Keele, & Tingley [27]. The direct acyclic graph representing the causal structure of this analysis is presented in Supporting Figure 1. In brief, this analysis decomposes the total effect between high‐dose corticosteroid treatment and all‐cause mortality into direct (i.e., condition where the mediator is held at its values associated with being untreated) and indirect effects (i.e., effects mediated through inflammation). This method contrasts effects using the potential‐outcomes framework, which averages the corresponding levels of counterfactual treatment exposure and mediators. This was accomplished by first regressing the selected biomarkers on high‐dose corticosteroid treatment using linear regression and regressing the probability of all‐cause mortality on the selected biomarkers using probit regression. We then estimated the total effect of high‐dose corticosteroid therapy on the probability of all‐cause mortality using probit regression, while controlling for these regressed mediation effects. These models allowed us to estimate the total effect, average direct effect, and average mediation effect, along with their 95% CI. These outputs were then used to estimate the % of total effect mediated. Parameter estimates were obtained using the “mediation” ado‐commands in Stata. In the models, including high‐dose corticosteroids, inverse probability of treatment weights (IPTW) were used to account for the time‐varying exposure, mediation and confounding occurring over time [28]. At each time‐point, the conditional probability of being treated was estimated from a logistic regression model, including the covariates P/F ratio (time‐updated), sex at birth (time‐fixed), age at ICU admission (time‐fixed), NLR (time‐updated), SOFA score (time‐updated), and time from admission duration (time‐updated). These probabilities were then recalculated to the probability of receiving the observed treatment, inversed and stabilized with the probability of receiving the observed treatment without covariates. To ensure that certain patients are not inadvertently over‐represented, weights were right‐truncated if they were above the 95th‐percentile. Results were unchanged if right‐truncation was ignored (data not shown).

To understand the concentration‐dependent relationship of mediation, we examined the association between high‐dose corticosteroids and mortality as levels of significantly mediating biomarkers increased in post hoc analysis. The probability of mortality was modeled using probit regression with variance estimations clustered for patient. High‐dose corticosteroids, tertiles of a given inflammatory biomarker and the interaction between the two were included as covariates. The decision to use tertiles was data‐driven and not based on clinically‐relevant thresholds. From this model, stratum‐specific parameter estimates comparing the probability of mortality between those who did vs. did not receive high‐dose corticosteroids and their 95% CI were calculated for each tertile of a given biomarker. The interaction between high‐dose corticosteroids and inflammation was tested on the interaction covariate using a Wald *χ* ^2^ test. These models used IPTWs to account for time‐varying confounding.

Statistical analyses were carried out in Stata (v18.0, College Station, TX). *p* < 0.05 was considered statistically significant. As these analyses were considered exploratory [29], we did not adjust *p*‐values for multiple comparisons. For all models, observations with missing data on any the covariables were removed.

### 2.4 Sensitivity Analysis

Several sensitivity analyses were conducted to assess certain assumptions of the model. First, we carried out a mediation analysis in which exposure to high‐dose corticosteroids was lagged by 1 day instead of current high‐dose use. Any departure of this analysis from the original analysis would suggest reverse causation. Second, patients who died within 2 days after baseline were excluded. Immediate death could have been more frequent in the high‐dose corticosteroid group than the standard‐dose group, inducing a differential selection bias. We carried out the mediation analysis while including all those with early death. Third, this analysis assumes that at each time point, conditional on the past, the relationships between exposure‐outcome, mediator‐outcome, and exposure‐mediator are unconfounded (i.e., sequential ignorability assumption). Residual confounding from unmeasured variables could violate this assumption. Mediational E‐values were then calculated to determine the minimum strength of association a confounder would need to have in the confounder‐outcome relations to explain away the direct or indirect effects [30].

## 3 Results

### 3.1 Description of the Study Population

From January 4, 2020, to December 31, 2022, 330 patients with COVID‐19 were admitted to the ICU with ARDS who fulfilled eligibility criteria. Three patients were excluded because they died within 2 days after ICU admission. In total, 327 were included in analysis, 122 (37.3%) of whom received high‐dose corticosteroid therapy.

Patient characteristics at baseline are shown in Table 1. The median age at baseline was 63 years (IQR = 55–70), median BMI was 29.1 kg/m^2^ (IQR = 25.8–32.3), and 74.9% of patients were male. As shown in Table 1, males and current smokers were more likely to have received high‐dose corticosteroid therapy during follow‐up. There were no significant differences in comorbidities and median levels of biomarkers at baseline between the two groups.

**TABLE 1:** Patient characteristics at baseline, stratified by high‐dose corticosteroids use during follow‐up.

Patient characteristics	All patients	During follow‐up		*p*
		No high‐dose corticosteroids	High‐dose corticosteroids	
	(*n* = 327)	(*n* = 205)	(*n* = 122)	
**Demographic**				
Age, years	63 (55–70)	62 (54–70)	65 (58–70)	0.16
Sex at birth, male	245 (74.9%)	143 (69.8%)	102 (83.6%)	**< 0.005**
BMI, kg/m^2^	29.1 (25.8–32.3)	29.4 (26.3–32.3)	28.3 (25.1–32.4)	0.29
Current smoking	8 (5.8%)	4 (4.2%)	4 (9.5%)	**< 0.001**
**Comorbidities**				
Peripheral vascular disease	12 (3.7%)	9 (4.4%)	3 (2.5%)	0.55
Pulmonary disease	27 (10.0%)	19 (10.7%)	8 (8.6%)	0.67
Neurological disease	13 (4.7%)	10 (5.6%)	3 (3.2%)	0.55
Renal disease	24 (8.8%)	14 (7.8%)	10 (10.5%)	0.50
Diabetes mellitus	58 (21.4%)	35 (19.9%)	23 (24.2%)	0.44
Immunodeficiency	22 (8.1%)	13 (7.3%)	9 (9.7%)	0.49
Malignancy	25 (9.1%)	14 (7.8%)	11 (11.6%)	0.38
**Laboratory parameters**				
CRP, mg/L	126.3 (55.7–219.8)	129.8 (67.6–206.2)	117.9 (46.3–231.9)	0.41
d‐dimer, mg/L	1.52 (0.83–3.91)	1.36 (0.77–4.03)	1.65 (0.88–3.86)	0.74
Ferritin, mg/L	1119 (612–2030)	1100 (571–1932)	1161.5 (697–2316)	0.22
Leukocyte count, ×10^9^/L	9.5 (6.94–12.22)	9.3 (6.74–12.0)	9.74 (7.45–13.1)	0.19
IL‐6, pg/mL	85 (19.5–202)	56 (18–189)	103 (24–236)	0.10
LDH, U/L	408.5 (322–519)	397 (321–495)	436 (323–567)	0.12
NLR	10.28 (6.48–15.63)	9.64 (6.01–15.68)	11.39 (6.7–15.57)	0.21
PCT, ng/mL	0.36 (0.15–0.85)	0.36 (0.16–0.82)	0.37 (0.14–0.95)	0.96
**Disease severity**				
APACHE‐IV score	0.188 (0.117–0.308)	0.161 (0.098–0.297)	0.229 (0.142–324)	**0.005**
SOFA score	6 (5–8)	6 (5–8)	7 (6–8)	**0.04**
P/F ratio, kPA	20.44 (13.11–27.33)	23 (15.80–28.33)	17.66 (12.00–24.60)	**< 0.001**
Length of ICU stay, days	16 (8–28)	11 (7–22)	23 (14–36)	**< 0.001**
All‐cause mortality	87 (26.6%)	34 (16.6%)	53 (43.4%)	**< 0.001**

*Note:* All continuous variables are reported as median (IQR) and all categorical variables as counts and percentages. Patients who received and did not receive high‐dose corticosteroids during follow‐up were compared using a Mann–Whitney *U* test for continuous variables and Pearson's *χ* ^2^ test for categorical variables. *p*‐values with statistically significant differences are given in bold.

Missing data were found in the following number of participants (no high‐dose corticosteroid and high‐dose groups, respectively): BMI (*n* = 3, *n* = 2); smoking (*n* = 109, *n* = 80); pulmonary disease (*n* = 28, *n* = 29); neurological disease (*n* = 25, *n* = 27); renal disease (*n* = 26, *n* = 27); diabetes mellitus (*n* = 29, *n* = 27); immunodeficiency (*n* = 27, *n* = 29); malignancy (*n* = 25, *n* = 27); d‐dimer (*n* = 15, *n* = 60); ferritin (*n* = 12, *n* = 48), leukocyte count (*n* = 3, *n* = 1); IL‐6 (*n* = 19, *n* = 72); LDH (*n* = 1, *n* = 0); NLR (*n* = 44, *n* = 103); PCT (*n* = 14, *n* = 55); APACHE‐IV score (*n* = 21, *n* = 45); SOFA score (*n* = 1, *n* = 5); P/F ratio (*n* = 13, *n* = 35).

Abbreviations: BMI, body mass index; CRP, C‐reactive protein; IL‐6, interleukin‐6; LDH, lactate dehydrogenase; NLR, neutrophil‐to‐lymphocyte ratio; P/F ratio, PaO_2_/FiO_2_; APACHE, acute physiology, age, and chronic health evaluation.

### 3.2 Determinants of Commencing High‐Dose Corticosteroids

Of the 122 patients who were treated with high‐dose corticosteroid therapy, treatment was either commenced at baseline (*n* = 15, 12.3%) or a median of 8 days (IQR = 4–12) after baseline. In multivariable analysis (Table 2), a lower P/F ratio (*p* < 0.001) and higher NLR (*p* < 0.001) were independently associated with commencing high‐dose corticosteroid therapy after adjustment for age, sex at birth, and SOFA score.

**TABLE 2:** Determinants of receiving high‐dose corticosteroids (logistic regression).

Variables	Univariable			Multivariable		
	OR	95% CI	*p*	aOR	95% CI	*p*
P/F ratio (per log × 10)	0.68	(0.62–0.74)	**< 0.001**	0.69	(0.61–0.78)	**< 0.001**
Age (per year)	1.01	(0.99–1.03)	0.34	0.98	(0.96–1.00)	0.061
BMI (per kg/m^2^)	0.98	(0.93–1.03)	0.42			
Current smoking	1.42	(0.74–2.76)	0.30			
Male sex at birth	0.56	(0.28–1.10)	**0.093**	0.73	(0.36–1.48)	0.38
Peripheral vascular disease	0.85	(0.23–3.20)	0.81			
Pulmonary disease	0.86	(0.37–2.03)	0.74			
Neurological disease	0.56	(0.15–2.13)	0.40			
Renal disease	1.08	(0.49–2.38)	0.84			
Diabetes mellitus	1.19	(0.67–2.11)	0.55			
Immunodeficiency	1.53	(0.67–3.48)	0.32			
Malignancy	1.50	(0.71–3.17)	0.29			
CRP (per log_10_ mg/L)	1.81	(1.24–2.65)	**0.002**			
d‐dimer (per log_10_ mg/L)	1.29	(0.80–2.10)	0.30			
Ferritin (per log_10_ mg/L)	1.97	(1.12–3.45)	**0.018**			
Leukocyte count (per log_10_ × 10^9^/L)	1.06	(1.02–1.10)	**0.002**			
IL‐6 (per log_10_ pg/mL × 10)	2.39	(1.75–3.27)	**< 0.001**			
LDH (per log_10_, U/L)	1.96	(0.57–6.71)	0.29			
NLR (per unit)	1.04	(1.03–1.06)	**< 0.001**	1.03	(1.01–1.05)	**< 0.001**
SOFA score (per unit)	1.21	(1.13–1.31)	**< 0.001**	1.07	(0.99–1.16)	0.089
APACHE‐IV score (per unit)	5.83	(1.29–26.39)	**0.022**			
Length of ICU stay (per day)	1.02	(1.00–1.04)	**0.016**			

*Note:* The variables from univariable analysis that were considered in multivariable analysis are in bold. *p*‐values with statistically significant differences are given in bold. In multivariable analysis, initial tests on variance inflation factors (VIF) indicated no collinearity between significant variables (in univariable analysis), age and gender (range of VIF = 1.09–1.93). The APACHE‐IV score was not considered in the multivariable model as the parameter estimate was considered unstable (i.e., large effect size with large variance). The following variables were removed as they were no longer below *p* < 0.10: leukocyte count, *p* = 0.95; CRP, *p* = 0.87; length of ICU stay, *p* = 0.59; ferritin, *p* = 0.39; and IL‐6, *p* = 0.20. All variables presented in the multivariable analysis were included in the model.

Abbreviations: APACHE, acute physiology, age, and chronic health evaluation; CRP, C‐reactive protein; IL‐6, interleukin‐6; LDH, lactate dehydrogenase; NLR, neutrophil‐to‐lymphocyte ratio; P/F ratio, PaO2/FiO2 ratio; SOFA, sequential organ failure assessment.

### 3.3 Mediation Effect of Inflammatory Biomarkers

Patients were followed a median of 20 days (IQR = 11–32) after baseline, totaling 7780 person‐days of observation (including 3796 person‐days on high‐dose treatment and 3984 person‐days not on high‐dose treatment). During follow‐up, 87 (26.6%) patients died a median of 18 days (IQR = 10–32) after hospital admission. The incidence rate of death was higher in those who did vs. did not receive high‐dose corticosteroids (incidence rate = 0.54, 95% CI = 0.42–0.71 and 0.21, 95% CI = 0.15–0.29 per person‐month, respectively; *p* < 0.001).

**FIGURE 1:**
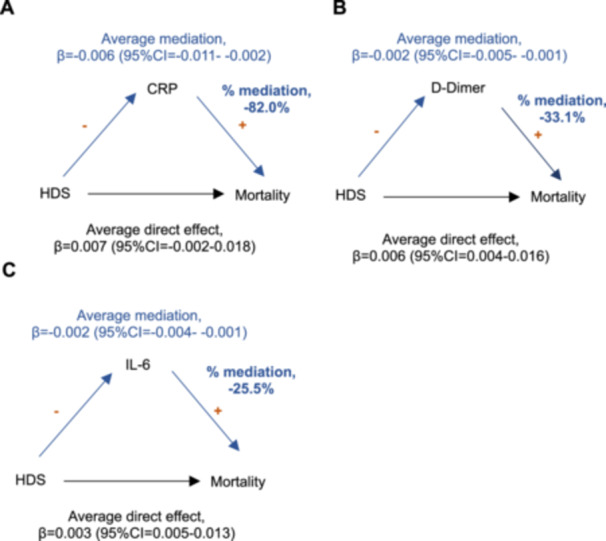
Inflammatory markers acting as mediators on the association between high‐dose corticosteroids use and mortality (causal mediation analysis). Main association between high‐dose corticosteroids (HDS) and mortality is depicted in black. The average direct effect here represents the association between HDS and mortality when the inflammatory marker is held at its values associated with being untreated. The mediation pathway is depicted in blue, while the directions of association between HDS and inflammatory markers and between inflammatory markers and mortality (while holding treatment constant) are depicted in orange (Supporting Tables 3 and 4, respectively). The “+” and “−” symbols represent positive and negative associations, respectively, between connected variables. The average indirect effect represents the effect mediated through inflammation and the % mediation is calculated by dividing the average mediation effects and the total effects. Negative mediation values indicate suppressive mediation, where direct and indirect effects have opposite directions. These parameter estimates represent multiple timepoints during the course of follow‐up—the underlying causal structure is depicted in Supporting Figure 1 in the form of a direct acyclic graph. These analyses included the following number of observations (*n*) and patients (*N*): CRP (A), *n* = 3357 and *N* = 314; d‐dimer (B), *n* = 3293 and *N* = 313; IL‐6 (C), *n* = 3309 and *N* = 312. Abbreviations: CI, confidence interval; CRP, C‐reactive protein; HDS, high‐dose corticosteroids; IL‐6, interleukin‐6.

The distribution of missing data from variables used in the causal mediation analysis is summarized in Supporting Table 2. Across all time points, the median IPTW used in the model was 0.78 (IQR = 0.53–1.29) for exposed and 0.80 (IQR = 0.73–0.94) for unexposed to high‐dose corticosteroids (the distribution of IPTW is given in Supporting Figure 2). Significant mediation (i.e., those biomarkers whose 95% CI of the average mediation effect did not cross 0) was found with CRP (Figure 1A), d‐dimer (Figure 1B), and IL‐6 (Figure 1C). The associations between high‐dose therapy and inflammatory markers and between inflammatory markers and mortality (while holding treatment constant) are given in Supporting Tables 3 and 4, respectively. There was no evidence that the other biomarkers were mediators (Supporting Figure 3). For CRP, d‐dimer, and IL‐6, lower levels reduced the effects between high‐dose corticosteroids and mortality. The strongest % of the total effect mediated by a given inflammatory marker was CRP (−82.0%), followed by d‐dimer (−33.1%), and IL‐6 (−25.5%).

Sensitivity analysis would indicate that reverse causation was unlikely across the three biomarkers with significant mediation (Supporting Table 5). There also appeared to be little differential selection bias when including the three individuals who died 2 days after commencing high‐dose corticosteroid therapy (Supporting Table 5). Finally, mediational E‐value analysis indicated that a minimal relative risk of an unmeasured confounder of the confounder‐outcome relation would need to be at least 2.66 (for CRP), 2.81 (for d‐dimer), and 2.05 (for IL‐6) to explain away the direct or indirect effects (Supporting Table 6).

### 3.4 Association Between High‐Dose Corticosteroids and Mortality at Various Level of Inflammation

**FIGURE 2:**
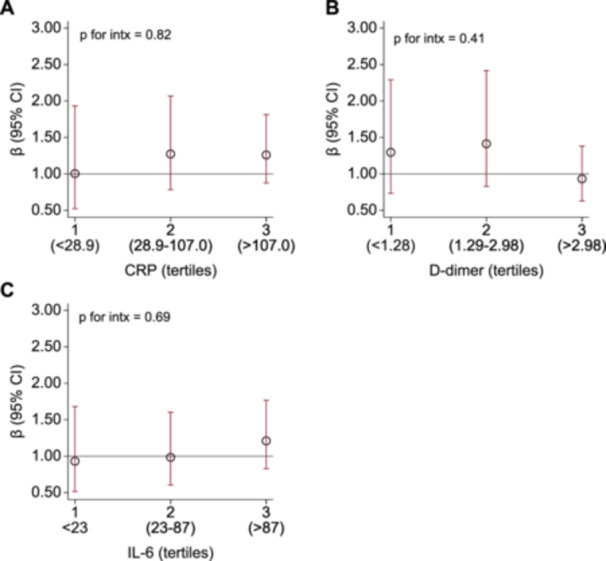
Association between high‐dose corticosteroids and mortality at levels of inflammation. Parameter estimates (in dots) and 95% CI (in capped lines) relatively comparing the probability of mortality between patients who did vs. did not receive high‐dose corticosteroids, stratified on quartiles of inflammatory marker levels during follow‐up. Only biomarkers demonstrating significant mediation were selected for analysis. (A) CRP, (B) D‐dimer, and (C) IL‐6. Abbreviations: CI, confidence interval; CRP, C‐reactive protein; HDS, high‐dose corticosteroids; IL‐6, interleukin‐6.

In post hoc analysis, we selected the biomarkers CRP, d‐dimer, and IL‐6 and examined their concentration‐dependent relationship on the association between high‐dose corticosteroids and mortality. As expected, the association between high‐dose corticosteroids and mortality increased as tertiles of CRP (Figure 2A) and IL‐6 (Figure 2C) increased (i.e., mediation effect is stronger as levels of CRP or IL‐6 decrease). In contrast, there was no clear change in association between high‐dose corticosteroids and mortality as tertiles of d‐dimer increased (Figure 2B). There was no significant interaction between high‐dose corticosteroids and tertiles of CRP, d‐dimer, or IL‐6 (*p* for interaction = 0.82, 0.41, and 0.69, respectively).

## 4 Discussion

In this analysis of patients admitted to the ICU with COVID‐19‐related ARDS, we observed that those who received high‐dose corticosteroids had a higher risk of mortality compared to patients who did not receive high‐dose corticosteroids, while accounting for time‐varying treatment exposure and mediation. It would appear that lower levels of inflammation induced by high‐dose corticosteroids would eliminate the risk of death from this treatment, confirming the hypothesis that inflammatory biomarkers mediate the association between high‐dose corticosteroids and mortality. It was nevertheless surprising that the mediation role of anti‐inflammation did not lead to reduced mortality.

To our knowledge, this is the first study to apply a causal mediation framework to investigate how inflammatory biomarkers mediate the association between high‐dose corticosteroids and mortality in patients with COVID‐19‐related ARDS. By using this approach, we demonstrated that the biomarkers CRP, d‐dimer, and IL‐6 significantly mediated the effect between high‐dose corticosteroids and mortality. The strongest mediation was observed with CRP. The risk of mortality decreased as levels of CRP, d‐dimer, and IL‐6 decreased. These results demonstrate the important role of inflammatory biomarkers when evaluating the effect of high‐dose corticosteroid therapy on mortality in COVID‐19‐related ARDS.

Inflammation is widely acknowledged as a key driver in the pathogenesis of non‐COVID‐19‐related ARDS [6, 7, 31]. Previous studies on ARDS have even identified certain subphenotypes of hyper‐inflammatory or “reactive” inflammation, which are characterized by excessive cytokine release and elevated levels of inflammatory biomarkers and are associated with higher rates of mortality [32, 33, 34]. In patients with COVID‐19‐related ARDS, the hyper‐inflammatory subphenotype appears to have a particularly decreased risk of mortality after treatment with corticosteroids at varying doses [35]. How these results are placed in the context of high‐dose corticosteroid therapy is somewhat elusive, as the inflammatory markers characterizing these profiles could vary differently during high‐dose corticosteroids.

The higher risk of mortality among patients receiving high‐dose corticosteroids could reflect residual indication bias or potential lack of treatment benefit. While our IPTW approach accounted for the time‐varying confounding with receiving treatment, unmeasured confounders inherent to observational studies may still influence these findings. The results from the E‐mediation analysis would however suggest unmeasured confounding to be unlikely and rather that HDS may be suboptimally effective or even harmful in this context. The lack of effectiveness could be due to inappropriate timing, inability to target the underlying pathophysiology of COVID‐19‐related ARDS, or treatment‐related complications. Evidence from non‐COVID‐19 ARDS studies has indeed shown that timing in this context is crucial, with early high‐dose corticosteroid therapy showing increased benefits on survival. In our study, HDS was initiated a median of 8 days after ICU admission, which is considered late in the course of ARDS. It should also be mentioned that many patients were transferred from other hospitals and some patients had already met ARDS criteria well before ICU admission (i.e., baseline in our study) [36, 37, 38]. Unfortunately, we did not collect data on potential complications, which cannot be evaluated in our study.

Our analysis identified three biomarkers that causally mediated the relationship between high‐dose corticosteroids and mortality. IL‐6, d‐dimer, and CRP showed similar directional effects, where decreasing levels were associated with reduced mortality. CRP is a well‐established biomarker of inflammation during critical conditions in the ICU, such as pneumonia [39, 40, 41], sepsis [42, 43], and more recently severe COVID‐19 [8, 9, 44, 45]. Higher levels of d‐dimer, a marker of thrombo‐embolic activity, have been strongly associated with poorer outcomes in COVID‐19, namely thrombosis, organ dysfunction, and mortality. Interestingly, compared with other respiratory viral infections, COVID‐19 is associated with a higher frequency and severity of clotting events [8, 10, 44, 46, 47, 48]. IL‐6, located upstream in the same pathway as CRP, is another important biomarker of disease progression and is also associated with poorer outcomes in severe inflammatory diseases [49, 50, 51, 52], including COVID‐19 [25, 44, 53, 54]. Interestingly, previous studies have shown that the combination of corticosteroids and IL‐6 receptor antagonists, such as tocilizumab, is associated with the greatest reduction in COVID‐19 mortality [55, 56, 57]. Importantly, CRP exhibited the strongest mediating effect. Subgroups of patients with COVID‐19‐related ARDS who are likely to have poorer outcomes during high‐dose corticosteroids could be identified using CRP and to a lesser extent, d‐dimer, or IL‐6.

These results need be interpreted within a certain context. We analyzed biomarkers of inflammation during the periods both prior to and after commencement of any type of high‐dose corticosteroid therapy. Prior to treatment, these biomarkers were elevated in the majority of patients. After commencing high‐dose corticosteroids, which occurred a median of 8 days after inclusion, not all patients showed decreases in inflammatory biomarkers [58]. Although there are clear survival benefits with the use of standard‐dose corticosteroids in patients with COVID‐19‐related ARDS [13], our observational study shows that administering high‐dose corticosteroids did not result in a further decrease in mortality. A potential explanation for the lack of benefit could be suboptimal timing of high‐dose corticosteroid administration, with treatment being initiated relatively late (i.e., a median of 8 days). Research suggests that initiating corticosteroid treatment within 3 days of ARDS diagnosis is most optimal for achieving beneficial outcomes in survival [59]. Alternatively, high‐dose corticosteroids may have been administered in patients whose ongoing inflammation was driven by other factors, such as ventilator‐associated pneumonia (VAP) or catheter‐related infections, which could have contributed to worsening clinical outcomes. Nevertheless, our results clearly indicate that if inflammatory biomarkers do not decrease as a result of high‐dose corticosteroid therapy, high‐dose corticosteroid treatment is highly ineffective and the risk of mortality increases.

Our study has several limitations. First, the absence of a standardized protocol for high‐dose corticosteroids during the early phase of the inclusion period means that the target population of those receiving high‐dose corticosteroids varied over time. This lack of uniform guidance led to substantial indication bias. Second, the causal analysis herein assumes no unmeasured confounding for the exposure‐outcome, exposure‐mediator, and mediator‐outcome relations. We did address this with the mediational E‐value analysis, and although unlikely, it cannot fully exclude unmeasured confounding. Third, the number of patients with COVID‐19‐related ARDS is currently limited, and therefore the generalizability of these findings could be restricted. However, we believe these results could still be relevant for other populations of individuals who develop ARDS. Finally, no data were available on co‐infections, leukocyte subpopulations, causes of death, or whether death occurred after hospital discharge, which could have strengthened the analysis on mortality. Furthermore, we did not have data on the reasons for hospital discharge, making it impossible to evaluate the types of competing risks that could have played a role in these results.

## 5 Conclusions

In conclusion, CRP, d‐dimer, and IL‐6 significantly mediated the association between receiving high‐dose corticosteroid and mortality. Importantly, while accounting for time‐varying exposure and mediation, patients who received high‐dose corticosteroids had an elevated risk of mortality. When inflammatory biomarkers CRP and IL‐6 are reduced, any deleterious effect of high‐dose corticosteroids is removed. These findings challenge the practice of dose escalation in patients with COVID‐19‐related ARDS. Therapeutic options, other than increasing corticosteroid dose, need to be evaluated, along with the role of mediation from inflammatory biomarkers during therapy.

## Author Contributions

All authors made a significant contribution to the work reported, whether that is in the conception, study design, execution, acquisition of data, analysis and interpretation, or in all these areas; took part in drafting, revising, or critically reviewing the article; gave final approval of the version to be published; have agreed on the journal to which the article has been submitted; and agree to be accountable for all aspects of the work.

## Ethics Statement

The study was approved by the local Medical Ethics Review Committee of the Erasmus University Medical Center under protocol number MEC‐2017‐417 and conducted according to the principles of the Declaration of Helsinki. Due to the urgency of conducting research in patients with COVID‐19, an exemption for consent was approved by the Medical Research Ethics Committee at the Erasmus University Medical Center. An opt‐out informed consent procedure (MEC‐2022‐0297) was used and patients who expressed objections to participation were excluded from the study.

## Consent

The authors have nothing to report.

## Conflicts of Interest

The authors declare no conflicts of interest.

## Supporting information


Supporting File 1

Supporting File 2

## Data Availability

The data that support the findings of this study are available from the corresponding author upon reasonable request.

## References

[1] G. Bellani , J. G. Laffey , T. Pham , et al., “Epidemiology, Patterns of Care, and Mortality for Patients With Acute Respiratory Distress Syndrome in Intensive Care Units in 50 Countries,” Journal of the American Medical Association 315 (2016): 788–800, 10.1001/jama.2016.0291. 26903337

[2] R. Chang , K. M. Elhusseiny , Y. C. Yeh , and W. Z. Sun , “COVID‐19 ICU and Mechanical Ventilation Patient Characteristics and Outcomes‐A Systematic Review and Meta‐Analysis,” PLoS One 16 (2021): e0246318, 10.1371/journal.pone.0246318. 33571301 PMC7877631

[3] A. H. Attaway , R. G. Scheraga , A. Bhimraj , M. Biehl , and U. Hatipoğlu , “Severe Covid‐19 Pneumonia: Pathogenesis and Clinical Management,” BMJ 372 (2021): n436, 10.1136/bmj.n436. 33692022

[4] M. P. Reddy , A. Subramaniam , C. Chua , et al., “Respiratory System Mechanics, Gas Exchange, and Outcomes in Mechanically Ventilated Patients With COVID‐19‐Related Acute Respiratory Distress Syndrome: A Systematic Review and Meta‐Analysis,” Lancet Respiratory Medicine 10 (2022): 1178–1188, 10.1016/S2213-2600(22)00393-9. 36335956 PMC9708089

[5] WHO Coronavirus (COVID‐19) Dashboard, accessed July 7, 2025, https://www.who.int/emergencies/diseases/novel-coronavirus-2019.

[6] S. Kaku , C. D. Nguyen , N. N. Htet , et al., “Acute Respiratory Distress Syndrome: Etiology, Pathogenesis, and Summary on Management,” Journal of Intensive Care Medicine 35 (2020): 723–737, 10.1177/0885066619855021. 31208266

[7] L. D. J. Bos and L. B. Ware , “Acute Respiratory Distress Syndrome: Causes, Pathophysiology, and Phenotypes,” Lancet 400 (2022): 1145–1156, 10.1016/S0140-6736(22)01485-4. 36070787

[8] D. Battaglini , M. Lopes‐Pacheco , H. C. Castro‐Faria‐Neto , P. Pelosi , and P. R. M. Rocco , “Laboratory Biomarkers for Diagnosis and Prognosis in COVID‐19,” Frontiers in Immunology 13 (2022): 857573, 10.3389/fimmu.2022.857573. 35572561 PMC9091347

[9] P. Malik , U. Patel , D. Mehta , et al., “Biomarkers and Outcomes of COVID‐19 Hospitalisations: Systematic Review and Meta‐Analysis,” BMJ Evidence‐Based Medicine 26 (2021): 107–108, 10.1136/bmjebm-2020-111536. PMC7493072 32934000

[10] S. Figliozzi , P. G. Masci , N. Ahmadi , et al., “Predictors of Adverse Prognosis in COVID‐19: A Systematic Review and Meta‐Analysis,” European Journal of Clinical Investigation 50 (2020): e13362, 10.1111/eci.13362. 32726868

[11] D. Annane , S. M. Pastores , B. Rochwerg , et al., “Guidelines for the Diagnosis and Management of Critical Illness‐Related Corticosteroid Insufficiency (CIRCI) in Critically Ill Patients (Part I): Society of Critical Care Medicine (SCCM) and European Society of Intensive Care Medicine (ESICM) 2017,” Intensive Care Medicine 43 (2017): 1751–1763. 28940011 10.1007/s00134-017-4919-5

[12] A. Agarwal , B. J. Hunt , M. Stegemann , et al., “A Living WHO Guideline on Drugs for Covid‐19,” BMJ 370 (2020): m3379, 10.1136/bmj.m3379. 32887691

[13] R. C. Group , P. Horby , W. S. Lim , et al., “Dexamethasone in Hospitalized Patients With Covid‐19,” New England Journal of Medicine 384 (2021): 693–704, 10.1056/NEJMoa2021436. 32678530 PMC7383595

[14] H. K. Siddiqi and M. R. Mehra , “COVID‐19 Illness in Native and Immunosuppressed States: A Clinical‐Therapeutic Staging Proposal,” Journal of Heart and Lung Transplantation 39 (2020): 405–407, 10.1016/j.healun.2020.03.012. PMC7118652 32362390

[15] R. Diagnostics , accessed January 5, 2024, https://diagnostics.roche.com/us/en/products/instruments/cobas-c-502-ins-2113.html.

[16] R. Diagnostics Roche Diagnostics, accessed January 5, 2014, https://diagnostics.roche.com/us/en/products/instruments/cobas-e-801-ins-2202.html.

[17] M. T. Beigmohammadi , L. Amoozadeh , F. Rezaei Motlagh , et al., “Mortality Predictive Value of APACHE II and SOFA Scores in COVID‐19 Patients in the Intensive Care Unit,” Canadian Respiratory Journal 2022 (2022): 5129314, 10.1155/2022/5129314. 35356088 PMC8958381

[18] B. Gallo Marin , G. Aghagoli , K. Lavine , et al., “Predictors of COVID‐19 Severity: A Literature Review,” Reviews in Medical Virology 31 (2021): 1–10, 10.1002/rmv.2146. PMC7855377 32845042

[19] C. Huang , J. Soleimani , S. Herasevich , et al., “Clinical Characteristics, Treatment, and Outcomes of Critically Ill Patients With COVID‐19: A Scoping Review,” Mayo Clinic Proceedings 96 (2021): 183–202, 10.1016/j.mayocp.2020.10.022. 33413817 PMC7586927

[20] L. Wynants , B. Van Calster , G. S. Collins , et al., “Prediction Models for Diagnosis and Prognosis of Covid‐19: Systematic Review and Critical Appraisal,” BMJ 369 (2020): m1328, 10.1136/bmj.m1328. 32265220 PMC7222643

[21] J. Vandenbrande , L. Verbrugge , L. Bruckers , et al., “Validation of the Acute Physiology and Chronic Health Evaluation (APACHE) II and IV Score in COVID‐19 Patients,” Critical Care Research and Practice 2021 (2021): 5443083, 10.1155/2021/5443083. 34258059 PMC8225448

[22] V. Sungono , H. Hariyanto , T. E. B. Soesilo , et al., “Cohort Study of the APACHE II Score and Mortality for Different Types of Intensive Care Unit Patients,” Postgraduate Medical Journal 98 (2022): 914–918, 10.1136/postgradmedj-2021-140376. 37063012

[23] A. Izcovich , M. A. Ragusa , F. Tortosa , et al., “Prognostic Factors for Severity and Mortality in Patients Infected With COVID‐19: A Systematic Review,” PLoS One 15 (2020): e0241955, 10.1371/journal.pone.0241955. 33201896 PMC7671522

[24] G. B. D. D. Collaborators , “Global Age‐Sex‐Specific Mortality, Life Expectancy, and Population Estimates in 204 Countries and Territories and 811 Subnational Locations, 1950–2021, and the Impact of the COVID‐19 Pandemic: A Comprehensive Demographic Analysis for the Global Burden of Disease Study 2021,” Lancet 403 (2024): 1989–2056, 10.1016/S0140-6736(24)00476-8. 38484753 PMC11126395

[25] J. N. Lieberum , S. Kaiser , J. Kalbhenn , H. Bürkle , and N. Schallner , “Predictive Markers Related to Local and Systemic Inflammation in Severe COVID‐19‐Associated ARDS: A Prospective Single‐Center Analysis,” BMC Infectious Diseases 23 (2023): 19, 10.1186/s12879-023-07980-z. 36631778 PMC9832419

[26] J. van Paassen , J. S. Vos , E. M. Hoekstra , K. M. I. Neumann , P. C. Boot , and S. M. Arbous , “Corticosteroid Use in COVID‐19 Patients: A Systematic Review and Meta‐Analysis on Clinical Outcomes,” Critical Care 24 (2020): 696. 10.1186/s13054-020-03400-9. 33317589 PMC7735177

[27] K. Imai , L. Keele , and D. Tingley , “A General Approach to Causal Mediation Analysis,” Psychological Methods 15 (2010): 309–334, 10.1037/a0020761. 20954780

[28] T. J. VanderWeele and E. J. Tchetgen Tchetgen , “Mediation Analysis With Time Varying Exposures and Mediators,” Journal of the Royal Statistical Society Series B: Statistical Methodology 79 (2017): 917–938, 10.1111/rssb.12194. 28824285 PMC5560424

[29] K. J. Rothman , “No Adjustments Are Needed for Multiple Comparisons,” Epidemiology 1 (1990): 43–46. January 1, 1990. 2081237

[30] L. H. Smith and T. J. VanderWeele , “Mediational E‐Values Approximate Sensitivity Analysis for Unmeasured Mediator‐Outcome Confounding,” Epidemiology 30 (2019): 835–837, 10.1097/Ede.0000000000001064. 31348008 PMC6768718

[31] M. A. Matthay , L. B. Ware , and G. A. Zimmerman , “The Acute Respiratory Distress Syndrome,” Journal of Clinical Investigation 122 (2012): 2731–2740, 10.1172/JCI60331. 22850883 PMC3408735

[32] C. S. Calfee , K. Delucchi , P. E. Parsons , B. T. Thompson , L. B. Ware , and M. A. Matthay , “Subphenotypes in Acute Respiratory Distress Syndrome: Latent Class Analysis of Data From Two Randomised Controlled Trials,” Lancet Respiratory Medicine 2 (2014): 611–620, 10.1016/S2213-2600(14)70097-9. 24853585 PMC4154544

[33] C. S. Calfee , K. L. Delucchi , P. Sinha , et al., “Acute Respiratory Distress Syndrome Subphenotypes and Differential Response to Simvastatin: Secondary Analysis of a Randomised Controlled Trial,” Lancet Respiratory Medicine 6 (2018): 691–698, 10.1016/S2213-2600(18)30177-2. 30078618 PMC6201750

[34] L. D. Bos , L. R. Schouten , L. A. van Vught , et al., “Identification and Validation of Distinct Biological Phenotypes in Patients With Acute Respiratory Distress Syndrome by Cluster Analysis,” Thorax 72 (2017): 876–883, 10.1136/thoraxjnl-2016-209719. 28450529 PMC5964254

[35] P. Sinha , D. Furfaro , M. J. Cummings , et al., “Latent Class Analysis Reveals COVID‐19‐related Acute Respiratory Distress Syndrome Subgroups With Differential Responses to Corticosteroids,” American Journal of Respiratory and Critical Care Medicine 204 (2021): 1274–1285, 10.1164/rccm.202105-1302OC. 34543591 PMC8786071

[36] K. P. Steinberg , L. D. Hudson , R. B. Goodman , et al., “Efficacy and Safety of Corticosteroids for Persistent Acute Respiratory Distress Syndrome,” New England Journal of Medicine 354 (2006): 1671–1684, 10.1056/NEJMoa051693. 16625008

[37] G. U. Meduri , A. S. Headley , E. Golden , et al., “Effect of Prolonged Methylprednisolone Therapy in Unresolving Acute Respiratory Distress Syndrome: A Randomized Controlled Trial,” Journal of the American Medical Association 280 (1998): 159–165, 10.1001/jama.280.2.159. 9669790

[38] G. U. Meduri , E. Golden , A. X. Freire , et al., “Methylprednisolone Infusion in Early Severe ARDS,” Chest 131 (2007): 954–963, 10.1378/chest.06-2100. 17426195

[39] M. Doganci , G. Eraslan Doganay , H. Sazak , et al., “The Utility of C‐Reactive Protein, Procalcitonin, and Leukocyte Values in Predicting the Prognosis of Patients With Pneumosepsis and Septic Shock,” Medicina (Kaunas, Lithuania) 60 (2024): 1560, 10.3390/medicina60101560. 39459346 PMC11509754

[40] Q. Liu , G. Sun , and L. Huang , “Association of the NLR, BNP, PCT, CRP, and D‐D With the Severity of Community‐Acquired Pneumonia in Older Adults,” Clinical Laboratory 69 (2023: 2416, 10.7754/Clin.Lab.2023.220330. 38084688

[41] W. Zhang , H. Xiao , X. Tong , L. He , X. Xu , and J. Dong , “Study on the Clinical Characteristics, Treatment, and Outcome Influencing Factors of Severe Pneumonia Complicated With ARDS,” Medicine 103 (2024): e40316, 10.1097/MD.0000000000040316. 39533637 PMC11557005

[42] T. Tian , B. Wei , and J. Wang , “Study of C‐Reactive Protein, Procalcitonin, and Immunocyte Ratios in 194 Patients With Sepsis,” BMC Emergency Medicine 21 (2021): 81. 10.1186/s12873-021-00477-5. 34233608 PMC8265098

[43] H. Koozi , M. Lengquist , and A. Frigyesi , “C‐Reactive Protein as a Prognostic Factor in Intensive Care Admissions for Sepsis: A Swedish Multicenter Study,” Journal of Critical Care 56 (2020): 73–79, 10.1016/j.jcrc.2019.12.009. 31855709

[44] L. Z. Hong , Z. X. Shou , D. M. Zheng , and X. Jin , “The Most Important Biomarker Associated With Coagulation and Inflammation Among COVID‐19 Patients,” Molecular and Cellular Biochemistry 476 (2021): 2877–2885. 10.1007/s11010-021-04122-4. 33742367 PMC7978444

[45] A. Kukoč , A. Mihelčić , I. Miko , et al., “Clinical and Laboratory Predictors at ICU Admission Affecting Course of Illness and Mortality Rates in a Tertiary COVID‐19 Center,” Heart & Lung 53 (2022): 1–10, 10.1016/j.hrtlng.2022.01.013. 35104727 PMC8784621

[46] N. Tang , D. Li , X. Wang , and Z. Sun , “Abnormal Coagulation Parameters Are Associated With Poor Prognosis in Patients With Novel Coronavirus Pneumonia,” Journal of Thrombosis and Haemostasis 18 (2020): 844–847, 10.1111/jth.14768. 32073213 PMC7166509

[47] J. D. McFadyen , H. Stevens , and K. Peter , “The Emerging Threat of (Micro)Thrombosis in COVID‐19 and Its Therapeutic Implications,” Circulation Research 127 (2020): 571–587, 10.1161/CIRCRESAHA.120.317447. 32586214 PMC7386875

[48] E. J. Nossent , A. R. Schuurman , T. D. Y. Reijnders , et al., “Pulmonary Procoagulant and Innate Immune Responses in Critically Ill COVID‐19 Patients,” Frontiers in Immunology 12 (2021): 664209, 10.3389/fimmu.2021.664209. 34054832 PMC8160522

[49] J. Song , D. W. Park , S. Moon , et al., “Diagnostic and Prognostic Value of Interleukin‐6, Pentraxin 3, and Procalcitonin Levels Among Sepsis and Septic Shock Patients: A Prospective Controlled Study According to the Sepsis‐3 Definitions,” BMC Infectious Diseases 19 (2019): 968. 10.1186/s12879-019-4618-7. 31718563 PMC6852730

[50] Y. Zhou , Y. Feng , X. Liang , et al., “Elevations in Presepsin, PCT, hs‐CRP, and IL‐6 Levels Predict Mortality Among Septic Patients in the ICU,” Journal of Leukocyte Biology 116 (2024): 890–900, 10.1093/jleuko/qiae121. 38776408

[51] R. S. Hotchkiss , L. L. Moldawer , S. M. Opal , K. Reinhart , I. R. Turnbull , and J. L. Vincent , “Sepsis and Septic Shock,” Nature Reviews Disease Primers 2 (2016): 16045, 10.1038/nrdp.2016.45. PMC5538252 28117397

[52] S. Kang and T. Kishimoto , “Interplay Between Interleukin‐6 Signaling and the Vascular Endothelium in Cytokine Storms,” Experimental & Molecular Medicine 53 (2021): 1116–1123. 10.1038/s12276-021-00649-0. 34253862 PMC8273570

[53] B. M. Henry , M. H. S. de Oliveira , S. Benoit , M. Plebani , and G. Lippi , “Hematologic, Biochemical and Immune Biomarker Abnormalities Associated With Severe Illness and Mortality in Coronavirus Disease 2019 (COVID‐19): A Meta‐Analysis,” Clinical Chemistry and Laboratory Medicine (CCLM) 58 (2020): 1021–1028, 10.1515/cclm-2020-0369. 32286245

[54] H. Akbari , R. Tabrizi , K. B. Lankarani , et al., “The Role of Cytokine Profile and Lymphocyte Subsets in the Severity of Coronavirus Disease 2019 (COVID‐19): A Systematic Review and Meta‐Analysis,” Life Sciences 258 (2020): 118167, 10.1016/j.lfs.2020.118167. 32735885 PMC7387997

[55] J. I. Iglesias , A. V. Vassallo , J. B. Sullivan , et al., “Retrospective Analysis of Anti‐Inflammatory Therapies During the First Wave of COVID‐19 at a Community Hospital,” World Journal of Critical Care Medicine 10 (2021): 244–259, 10.5492/wjccm.v10.i5.244. 34616660 PMC8462025

[56] R.‐C. Investigators , A. C. Gordon , P. R. Mouncey , et al., “Interleukin‐6 Receptor Antagonists in Critically Ill Patients With Covid‐19,” New England Journal of Medicine 384 (2021): 1491–1502, 10.1056/NEJMoa2100433. 33631065 PMC7953461

[57] O. Abani , A. Abbas , F. Abbas , et al., “Tocilizumab in Patients Admitted to Hospital With COVID‐19 (RECOVERY): A Randomised, Controlled, Open‐Label, Platform Trial,” Lancet 397 (2021): 1637–1645, 10.1016/S0140-6736(21)00676-0. 33933206 PMC8084355

[58] K. Daenen , A. Boyd , J. Huijben , et al., “Inflammatory Biomarkers Demonstrate Predictive Capacity for Mortality in COVID‐19‐Related ARDS Patients Receiving High‐Dose Corticosteroids: A Longitudinal Analysis,” Journal of Inflammation Research 18 (2025): 2395–2408, 10.2147/JIR.S502188. 39991661 PMC11846612

[59] Y. Hirano , S. Madokoro , Y. Kondo , K. Okamoto , and H. Tanaka , “Corticosteroid Treatment for Early Acute Respiratory Distress Syndrome: A Systematic Review and Meta‐Analysis of Randomized Trials,” Journal of Intensive Care 8 (2020): 91, 10.1186/s40560-020-00510-y. 33722302 PMC7720037

