# Supplementary material for: Inflammatory Biomarkers as Mediators of the Effect Between High‐Dose Corticosteroid Therapy and Mortality in COVID‐19‐Related ARDS: A Causal Mediation Analysis
Source: Immun Inflamm Dis. 2026 Apr 26;14(4):e70427. doi: 10.1002/iid3.70427 (PMC13111805; doi:10.1002/iid3.70427)
Supplement: Supplementary file 1 — Supporting File 1 [file IID3-14-e70427-s002.docx]

**SUPPLEMENTARY MATERIALS**

**Inflammatory biomarkers as mediators of the effect between high-dose corticosteroid therapy and mortality in COVID-19-related ARDS: a causal mediation analysis**

**Table of contents**

[SUPPLEMENTARY TABLES 2](#_Toc212126018)

[Supplementary Table 1. Equivalent corticosteroids and dosages (total daily dosing) 2](#_Toc212126019)

[Supplementary Table 2. Missingness of variables used in the causal mediation analysis 3](#_Toc212126020)

[Supplementary Table 3. Association between high-dose corticosteroid treatment on inflammatory biomarkers (linear regression) 4](#_Toc212126021)

[Supplementary Table 4. Association between inflammatory biomarkers and mortality (probit regression) 5](#_Toc212126022)

[Supplementary Table 5. Sensitivity analyses of causal mediation analysis 6](#_Toc212126023)

[Supplementary Table 6. E-value analysis for the confounder-outcome relation 7](#_Toc212126024)

[SUPPLEMENTARY FIGURES 8](#_Toc212126025)

[Supplementary Figure 1. Direct acyclic graph depicting the causal mediation analysis with time-varying mediation on inflammatory markers 8](#_Toc212126026)

[Supplementary Figure 2. Distribution of inverse probability of treatment weights used in the causal mediation analysis 9](#_Toc212126027)

[Supplementary Figure 3. Inflammatory markers unlikely to act as mediators on the association between high-dose corticosteroids use and mortality (causal mediation analysis) 10](#_Toc212126028)

[SUPPLEMENTARY REFERENCES 11](#_Toc212126029)

# SUPPLEMENTARY TABLES

## Supplementary Table 1. Equivalent corticosteroids and dosages (total daily dosing)

| Drug | Equivalent dose (mg) | Low-dose (mg) | High-dose (mg) |
| --- | --- | --- | --- |
| Dexamethasone | 0.75 | ≤6 | >6 |
| Cortisone | 25 | ≤200 | >200 |
| Hydrocortisone | 20 | ≤160 | >160 |
| Prednis(ol)one | 5 | ≤40 | >40 |
| Methylprednisolone | 4 | ≤32 | >32 |
| Betamethasone | 0.75 | ≤6 | >6 |

This table represents commonly used corticosteroids and their dosages. Equivalent dose is derived from Supplementary reference [1] (in Dutch).

## Supplementary Table 2. Missingness of variables used in the causal mediation analysis

| Variable | Observations (*N*=7,287) | | Patients (*N*=327) | |
| --- | --- | --- | --- | --- |
|  | *n* | (%) | *n* | (%) |
| P/F ratio | 5917 | (81.2) | 320 | (97.9) |
| Age | 7287 | (100) | 327 | (100) |
| Sex at birth | 7287 | (100) | 327 | (100) |
| SOFA score | 6574 | (90.2) | 327 | (100) |
| CRP | 7287 | (100) | 327 | (100) |
| D-dimer | 5641 | (77.4) | 326 | (99.7) |
| Ferritin | 5797 | (79.6) | 326 | (99.7) |
| Leukocyte count | 7239 | (99.3) | 327 | (100) |
| IL-6 | 5452 | (74.8) | 324 | (99.1) |
| LDH | 7189 | (98.7) | 327 | (100) |
| NLR | 3931 | (53.9) | 323 | (98.8) |

Number of observations are derived from the total number of observations from all patients eligible for analysis. The *n* and % represent the number of observations with data and the percent of observations with available data, respectively, on a given variable. Only variables used in the causal mediation analysis are considered.

Abbreviations: P/F ratio, PaO2/FiO2 ratio; CRP, C-reactive protein; IL-6, interleukin-6; LDH, lactate dehydrogenase; NLR, neutrophil-to-lymphocyte ratio; SOFA, Sequential Organ Failure Assessment.

## Supplementary Table 3. Association between high-dose corticosteroid treatment on inflammatory biomarkers (linear regression)

| Biomarker | β (95%CI) | *p* |
| --- | --- | --- |
| CRP (difference in log_10_ mg/L) | -0.33 (-0.44, -0.22) | <0.001 |
| D-dimer (difference in log_10_ mg/L) | -0.12 (-0.20, -0.03) | 0.006 |
| Ferritin (difference in log_10_ mg/L) | -0.096 (-0.15, 0.03) | 0.17 |
| Leukocyte count (difference in x10^9^/L) | -0.41 (-1.44, 0.62) | 0.44 |
| IL-6 (difference in log_10_ pg/mL) | -0.18 (-0.33, -0.04) | 0.014 |
| LDH (difference in log_10_ U/L) | -0.01 (-0.04, 0.02) | 0.61 |
| NLR (difference in value) | 1.29 (-0.44, 3.03) | 0.14 |

Coefficients represent the difference in average levels of biomarkers between those who received and did not receive high-dose corticosteroid treatment, and are reported with their 95%CI.

Abbreviations: CRP, C-reactive protein; IL-6, interleukin-6; LDH, lactate dehydrogenase; NLR, neutrophil-to-lymphocyte ratio

## Supplementary Table 4. Association between inflammatory biomarkers and mortality (probit regression)

| Biomarker | β (95%CI) | *p* |
| --- | --- | --- |
| CRP (per log_10_ mg/L) | 0.49 (0.19, 0.79) | 0.001 |
| D-dimer (per log_10_ mg/L) | 0.54 (0.23,0.84) | 0.001 |
| Ferritin, (per log_10_ mg/L) | 0.36 (0.04, 0.68) | 0.03 |
| Leukocyte count, (per x10^9^/L) | 0.05 (0.03, 0.07) | <0.001 |
| IL-6, (per log_10_ pg/mL) | 0.31 (0.10, 0.51) | 0.004 |
| LDH (per log_10_ U/L) | 1.06 (0.51, 1.60) | <0.001 |
| NLR (per value) | 0.011 (0.005, 0.018) | <0.001 |

Coefficients represent the exponentiated relative difference in probability of mortality for each unit increase in biomarkers, while holding levels of biomarkers constant, and are reported with their 95%CI.

Abbreviations: CI, confidence intervals; CRP, C-reactive protein; IL-6, interleukin-6; LDH, lactate dehydrogenase.

## Supplementary Table 5. Sensitivity analyses of causal mediation analysis

| **Inflammatory marker** | **Main analysis** | | **Sensitivity analysis** | | | |
| --- | --- | --- | --- | --- | --- | --- |
|  |  |  | **Lagged HDS exposure** | | **No exclusion of early death** | |
|  | **Average mediation effect** | **Average direct effect** | **Average mediation effect** | **Average direct effect** | **Average mediation effect** | **Average direct effect** |
| CRP, mg/L | -0.006 (-0.011, -0.002) | 0.007 (-0.002, 0.018) | -0.007 (-0.013, -0.003) | 0.008 (-0.02, 0.019) | -0.006 (-0.011, -0.002) | 0.007 (-0.003, 0.018) |
| D-dimer, mg/L | -0.002 (-0.005, -0.001) | 0.006 (-0.004, 0.016) | -0.002 (-0.005, -0.001) | 0.005 (-0.004, 0.016) | -0.002 (-0.005, -0.001) | 0.006 (-0.004, 0.016) |
| Ferritin, mg/L | -0.001 (-0.003, 0.000) | 0.003 (-0.005, 0.013) | -0.001 (-0.003, 0.000) | 0.003 (-0.006, 0.013) | -0.001 (-0.003, 0.000) | 0.003 (-0.006, 0.013) |
| Leukocyte count, x10^9^/L | -0.001 (-0.003, 0.001) | 0.003 (-0.005, 0.013) | -0.001 (-0.002, 0.001) | 0.003 (-0.006, 0.012) | -0.001 (-0.003, 0.001) | 0.003 (-0.005, 0.013) |
| IL-6, pg/mL | -0.002 (-0.004, -0.001) | 0.003 (-0.005, 0.013) | -0.002 (-0.005, -0.001) | 0.003 (-0.006, 0.013) | -0.002 (-0.004, -0.001) | 0.003 (-0.006, 0.013) |
| LDH, U/L | 0.000 (-0.002, 0.001) | 0.003 (-0.006, 0.013) | -0.001 (-0.002, 0.001) | 0.003 (-0.006, 0.012) | 0.000 (-0.002, 0.001) | 0.003 (-0.006, 0.013) |
| NLR | 0.001 (-0.001, 0.001) | 0.002 (-0.006, 0.012) | 0.001 (-0.001, 0.002) | 0.002 (-0.007, 0.011) | 0.001 (-0.001, 0.002) | 0.002 (-0.007, 0.012) |

*β* and their 95% confidence intervals are given for the average mediation and direct effects in the causal mediation analysis. This analysis examines inflammatory markers as mediators on the association between high-dose corticosteroid use and mortality. In this table, the main analysis are compared with two sensitivity analyses: lagged high-dose corticosteroid exposure (i.e., the exposure to high-dose therapy on a given day if replaced by exposure from the previous day) and including all those who died early (i.e., <2 days after admission to the intensive care unit).

Abbreviations: CRP, C-reactive protein; HDS, high-dose corticosteroids; IL-6, interleukin-6; LDH, lactate dehydrogenase; NLR, neutrophil-to-lymphocyte ratio.

## Supplementary Table 6. E-value analysis for the confounder-outcome relation

| Inflammatory marker | E-value |
| --- | --- |
| CRP, mg/L | 2.66 |
| D-dimer, mg/L | 2.81 |
| Ferritin, mg/L | 2.22 |
| Leukocyte count, x10^9^/L | 1.28 |
| IL-6, pg/mL | 2.05 |
| LDH, U/L | 5.20 |
| NLR | 1.12 |

The E-value represents the minimal strength of association of the confounder-outcome relation (as well as the approximate strength of the confounder-mediator relation) required to explain away a direct or indirect effect [2]. This calculation is based on the observed relative risk of the exposure-mediator and mediator-outcome associations, respectively. Given that the parameter estimate from the exposure-mediator association is from a linear regression model, the E-value for the confounder-mediator relation cannot be assessed. Instead, these values represent only the minimal strength of association of the confounder-outcome relation needed to explain away the direct or indirect effect. These values are calculated for each of the inflammatory markers studied.

Abbreviations: CRP, C-reactive protein; HDS, high-dose corticosteroids; IL-6, interleukin-6; LDH, lactate dehydrogenase; NLR, neutrophil-to-lymphocyte ratio.

# SUPPLEMENTARY FIGURES

## Supplementary Figure 1. Direct acyclic graph depicting the causal mediation analysis with time-varying mediation on inflammatory markers

…

*Y*

*HDS_2_*

*M_2_*

*L_2_*

*L_1_*

*M_1_*

*HDS_1_*

The graph above depicts the time-varying mediation effect during follow-up with *HDS_t_* representing high-dose corticosteroid therapy exposure at time *t*, *D* representing the outcome death, *M_t_* representing the mediating biomarker at level *m* at time *t*, and *L_t_* representing the vector of mediator-outcome confounders at time *t*. In our analysis, the confounders included are sex at birth (time-fixed), age at admission (time-fixed), neutrophil to lymphocyte ratio (time-updated), PaO2/FiO2 ratio (time-updated), Sequential Organ Failure Assessment score (time-updated), and time from admission duration (time-updated). We assume that no other biases between mediator-outcome confounders are present (e.g., collider-stratification bias) and that the assumptions of causal mediation analysis hold (e.g., positivity assumption of accounting for time-varying mediation, no unmeasured confounding on the average mediation effect and average direct effect). This graph follows the theoretical framework proposed in supplementary reference [3], while assuming no exposure-mediator confounders.

## Supplementary Figure 2. Distribution of inverse probability of treatment weights used in the causal mediation analysis

The distribution of the inverse probability of treatment weights (IPTW), right-truncated at the 95%tile, are depicted with a kernel-density plot. These are given for observations for which individuals were exposed or unexposed to high-dose corticosteroid (HDS) therapy. There is overlap between the weights, yet there is a much larger peak of unexposed observations at lower levels of the weight.

## Supplementary Figure 3. Inflammatory markers unlikely to act as mediators on the association between high-dose corticosteroids use and mortality (causal mediation analysis)

| **A** | HDS  Mortality  Ferritin  Average direct effect,  β=0.003 (95%CI=0.005-0.013)  Average mediation,  β=-0.001 (95%CI=-0.003-0.0004)  **% mediation,**  **-11.7%**  **-**  **+** | **B** | Mortality  HDS  Leukocytes  Average direct effect,  β=0.003 (95%CI=-0.005-0.013)  Average mediation,  β=-0.0006 (95%CI=-0.0025-0.0011)  **% mediation,**  **-9.9%**  **-**  **+** |
| --- | --- | --- | --- |
| **C** | Average direct effect,  β=0.003 (95%CI=-0.006-0.013)  LDH  **(-)**  **+**  **% mediation,**  **-4.2%**  Average mediation,  β=-0.001 (95%CI=-0.002-0.001)  HDS  Mortality |  | Average mediation,  β=0.0005 (95%CI=-0.0002-0.0015)  HDS  Mortality  NLR  Average direct effect,  β=0.006 (95%CI=-0.001-0.014)  **% mediation,**  **8.2%**  **(+)**  **+** |

Main association between high-dose corticosteroids (HDS) and mortality is depicted in black. The average direct effect here represents the association between HDS and mortality when the inflammatory marker is held at its values associated with being untreated. The mediation pathway is depicted in blue, while the directions of association between HDS and inflammatory markers and between inflammatory markers and mortality (while holding treatment constant) are depicted in orange (Supplementary Tables 3 and 4, respectively). : '+' and '−' symbols represent positive and negative associations, respectively, between connected variables. The average indirect effect represents the effect mediated through inflammation and the % mediation is calculated by dividing the average mediation effects and the total effects. Negative mediation values indicate suppressive mediation, where direct and indirect effects have opposite directions. These parameter estimates represent multiple timepoints during the course of follow-up—the underlying causal structure is depicted in Supplementary Figure 1 in the form of a direct acyclic graph. These analyses included the following number of observations (*n*) and patients (*N*): ferritin, *n*=3350 and *N*=314; leukocytes, *n*=3357 and *N*=314; LDH, *n*=3353 and *N*=314; NLR, *n*=3357 and *N*=314.

Abbreviations: CI, confidence interval; LDH, lactate dehydrogenase; NLR, neutrophil-to-lymphocyte ratio.

# SUPPLEMENTARY REFERENCES

1. Farmacotherapeutisch Kompas, Geneesmiddelgroepen. Geneesmiddelenoverzicht. Website: <https://www.farmacotherapeutischkompas.nl/bladeren/groepsteksten/corticosteroiden__systemisch>. [Accessed 23 October 2025]

2. Smith LH, VanderWeele TJ. Mediational E-values: Approximate Sensitivity Analysis for Unmeasured Mediator-Outcome Confounding. Epidemiology. 2019 Nov;30(6):835-837.

3. VanderWeele TJ, Tchetgen Tchetgen EJ. Mediation analysis with time varying exposures and mediators. J R Stat Soc Series B Stat Methodol. 2017 Jun;79(3):917-938.
